# Experimental model of peri-prosthetic infection of the knee caused by *Staphylococcus aureus* using biomaterials representative of modern TKA

**DOI:** 10.1242/bio.045203

**Published:** 2019-09-15

**Authors:** Jodie L. Morris, Hayley L. Letson, Andrea Grant, Matthew Wilkinson, Kaushik Hazratwala, Peter McEwen

**Affiliations:** 1 Orthopaedic Research Institute of Queensland, Townsville 4812, Australia; 2 College of Medicine, Division of Tropical Health and Medicine, James Cook University, Townsville 4811, Australia

**Keywords:** Animal model, Prosthetic joint infection, Biofilm, Inflammation, *Staphylococcus aureus*, Total knee arthroplasty

## Abstract

Prosthetic joint infection (PJI) following total knee arthroplasty (TKA) remains the leading cause for revision surgery, with *Staphylococcus aureus* the bacterium most frequently responsible. We describe a novel rat model of implant-associated *S. aureus* infection of the knee using orthopaedic materials relevant to modern TKA. Male Sprague-Dawley rats underwent unilateral knee implant surgery, which involved placement of a cementless, porous titanium implant into the femur, and an ultra-highly cross-linked polyethyelene (UHXLPE) implant into the proximal tibia within a mantle of gentamicin-laden bone cement. *S. aureus* biofilms were established on the surface of titanium implants prior to implantation into the femur of infected animals, whilst control animals received sterile implants. Compared to controls, the time taken to full weight-bear and recover pre-surgical body weight was greater in the infected group. Neutrophils and C-reactive protein levels were significantly higher in infected compared to control animals at day 5 post surgery, returning to baseline levels for the remainder of the 28-day experimental period. Blood cultures remained negative and additional plasma inflammatory markers were comparable for control and infected animals, consistent with the clinical presentation of delayed-onset PJI. *S. aureus* was recovered from joint tissue and implants at day 28 post surgery from all animals that received pre-seeded titanium implants, despite the use of antibiotic-laden cement. Persistent localised infection was associated with increased inflammatory responses and radiological changes in peri-implant tissue. The availability of a preclinical model that is reproducible based on the use of current TKA materials and consistent with clinical features of delayed-onset PJI will be valuable for evaluation of innovative therapeutic approaches.

## Abstract (precis)

**Summary:** The availability of a reproducible animal model using current and clinically representative materials will facilitate translational research for innovative therapeutic approaches for prosthetic joint infections caused by *Staphylococcus aureus*.

## INTRODUCTION

Currently, more than 4.7 million and 600,000 people in the USA and Australia, respectively, are estimated to be living with a total knee arthroplasty (TKA), with conservative projections estimating a 143% increase in incidence rates of TKA by 2050 (Maradit Kremers et al., 2015; AOANJRR, 2017; Inacio et al., 2017). Significant advancements to preoperative and surgical protocols and orthopaedic materials have reduced postoperative infection rates to less than 2%, however peri-prosthetic joint infection (PJI) remains the leading cause of implant failure following TKA (Springer et al., 2017; Tande and Patel, 2014). Methicillin-sensitive *Staphylococcus* *aureus* (MSSA) is the most common cause of PJI following TKA (Guo et al., 2017; Tande and Patel, 2014). Diagnosis and treatment of PJI poses a significant burden on both the patient and healthcare system, with eradication typically requiring multiple surgical interventions, prolonged hospitalisation, and aggressive and extensive antibiotic therapy (Beam and Osmon, 2018; King et al., 2018; Tande et al., 2017).

PJI are broadly classified according to the time from arthroplasty to development of infection (Beam and Osmon, 2018). Early-onset PJIs are defined as those occurring within 3 months of surgery, and arise due to intraoperative contamination with either a large bacterial burden or a virulent bacterial strain. Delayed-onset PJIs result from the introduction of less virulent microbes during surgery, and as such tend to become clinically apparent between 3 months to 1 year post surgery. In contrast, late-onset PJIs present more than 1 year post surgery and are frequently due to haematogenous seeding of the implanted joint from a distant site of infection. Delayed and late-onset PJIs typically involve implant-associated biofilms (Beam and Osmon, 2018).

Bio-inert orthopaedic materials such as titanium provide habitable substrates for biofilm formation, a growth state which serves to facilitate bacterial survival in hostile environments (Kostakioti et al., 2013; Ricciardi et al., 2018). Colonisation of an implant begins with adhesion of planktonic (free-floating) bacteria to the implant surface, upregulation of genes that facilitate a sessile lifestyle, proliferation and aggregation of bacterial cells into micro-colonies. The bacterial aggregates produce extracellular polymeric substances (EPS), at which point bacterial attachment becomes irreversible. Subsequent maturation of the biofilm is regulated by highly sophisticated, intercellular signalling networks and involves the development of a multi-layered, three-dimensional (3D) microbial community encased within a self-produced matrix of carbohydrate-rich polymers, proteins and nucleic acids (Ricciardi et al., 2018). Detachment and dispersal of planktonic bacterial cells from the periphery of mature biofilms facilitates dissemination and seeding of distant sites (Kostakioti et al., 2013). The metabolic activity of bacterial cells within a biofilm varies across a spectrum that inversely corresponds to nutrient availability, with those closest to the implant surface exhibiting metabolic inactivity and slower growth rates (Kostakioti et al., 2013). As such, implant-associated biofilms serve to protect bacteria from the host immune response and antibiotics, facilitating persistent infection and increased likelihood for the emergence of antibiotic-resistant bacterial strains (Arciola et al., 2018; Ricciardi et al., 2018). Often characteristic signs and symptoms of bacterial infection, such as elevated systemic inflammatory markers, are absent since bacteria within the biofilm are shielded from host immune responses (Beam and Osmon, 2018). Further, diagnosis of biofilm-associated PJI is also problematic due to the difficulty in removal and culture of bacterial cells from mature biofilms using conventional microbiological methods (Arciola et al., 2018; Beam and Osmon, 2018).

The difficulty preventing, diagnosing and eradicating biofilm-associated PJI, and the continued emergence of bacterial resistance to conventional and current antibiotics, is driving global interest in development of innovative therapeutic approaches (Li and Webster, 2018; Osmon, 2017). Clinically relevant small animal models are essential for preclinical evaluation of new preventative and therapeutic strategies. Several rodent models of implant-related *S. aureus* osteomyelitis have been described (Edelstein et al., 2017; Lucke et al., 2003; Reizner et al., 2014; Schindeler et al., 2018; Søe et al., 2013). However, many are based on the use of orthopaedic materials that do not reflect current clinical practice for arthroplasty, thus potentially limiting the translational capacity of findings. To address this, we sought to develop an experimental model of delayed-onset PJI caused by MSSA using current and clinically relevant TKA biomaterials.

In modern TKA, hybrid fixation techniques involving a cementless femoral component and a cemented tibial component is often used (Vertullo et al., 2018). Titanium alloys are one of the most commonly used metals in non-bearing surfaces of orthopaedic implants, whilst ultra-highly cross-linked polyethyelene (UHXLPE) is used for articulating surfaces (Bravin and Dietz, 2018). To reflect this combination of biomaterials and surgical techniques, we developed a rat model of knee implant surgery using a 3D-printed porous titanium implant that is press-fit into the femur, and a cemented UHXLPE tibial implant. We then progressed this surgical model to one representative of delayed-onset PJI caused by MSSA, using a previously characterised clinical strain from a patient with post-TKA PJI. The biofilm-forming capacity of the MSSA strain on the titanium implants used in the current study was recently demonstrated (Morris et al., 2018). Bacterial surface adhesion and irreversible attachment is a pivotal step in implant colonisation and establishment of persistent infection (Arciola et al., 2018). To ensure consistency in the number of implant-adherent bacteria, and therefore consistency and reliability of the experimental infection model, titanium scaffolds were pre-seeded with MSSA prior to implantation. Akin to the features of delayed-onset PJI, we demonstrate the establishment of a persistent infection that is localised to the implanted knee in all animals at 4 weeks after surgery, in the absence of bacteraemia and systemic inflammation.

## RESULTS

### Clinical outcomes

**Fig. 1.**
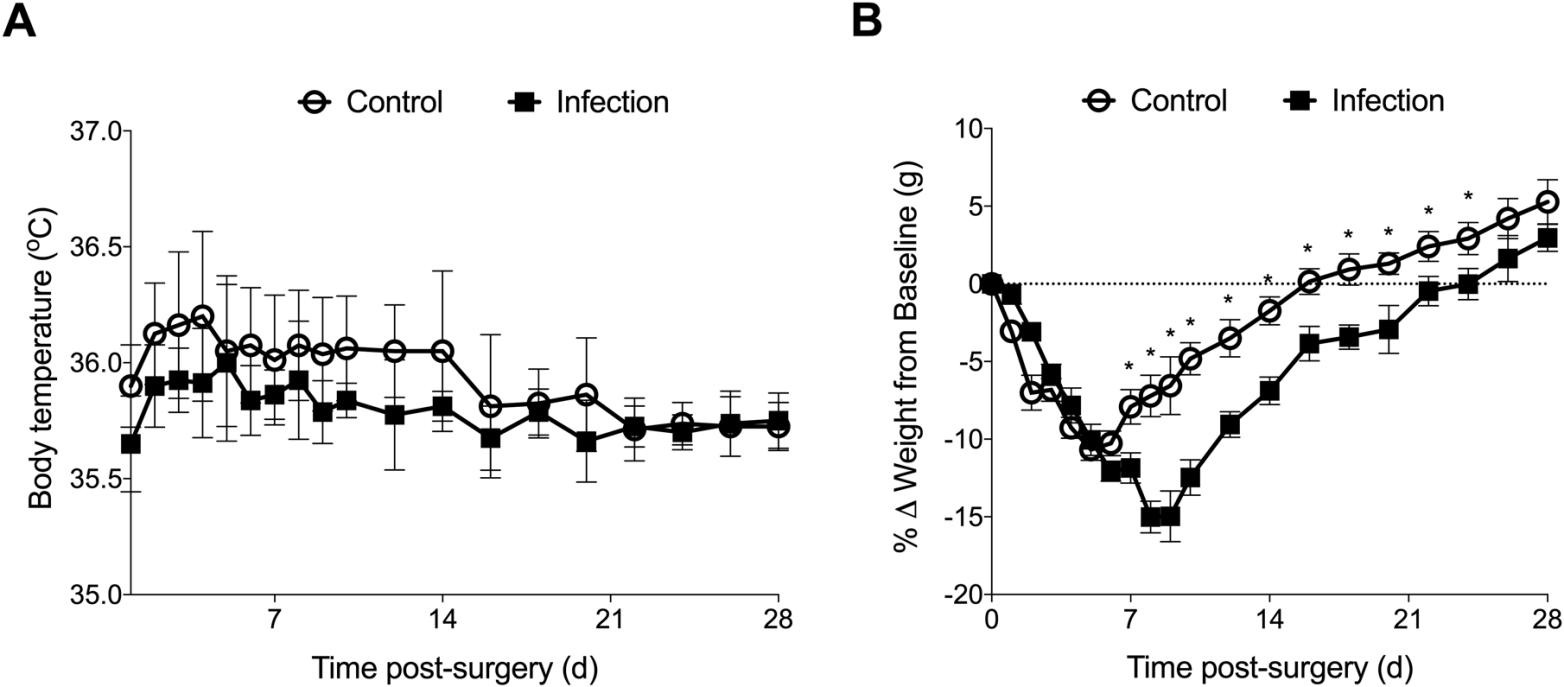
**Changes in body temperature and weight in control (*n*=12) and infected (*n*=13) animals following knee implant surgery.** Data show mean±s.e.m. *P<0.05 compared to control animals, two-way ANOVA with Holm-Sidak multiple comparison test.

Radiographs at day 7 post surgery confirmed the titanium and polyethylene implants were appropriately seated and stable within the femoral and tibial canals, respectively (Fig. S1A). All animals survived surgery and the postoperative period, with no signs of systemic illness. Based on improved clinical scores, pain relief was ceased for all animals by day 5 post surgery with no adverse clinical effects observed through the remainder of the experimental period. No significant differences were observed between body temperatures of control and infected animals throughout the experimental period (Fig. 1A). Control animals were able to partially bear weight on the operated limb within 48 h, with the median time to full weight-bearing 4 days post surgery (range, 3–6 days). In contrast, while *S. aureus-*infected animals were able to bear partial weight within 48 h of surgery, the median time to full weight-bear was significantly greater than control animals (4 versus 26 days post surgery), with four of eight animals not returning to full weight-bearing within the 28-day experimental period (*P*<0.001). Despite minor weight loss in the first week following surgery, all control animals returned to and exceeded pre-surgical weights within 21 days post surgery (Fig. 1B). In contrast, the time taken for animals in the infected group to return to pre-surgical body weight was delayed (control, 16.9±4.6 versus infection, 23.3±5.0 days post surgery, *P*=0.019; Fig. 1B).

### Haematology and systemic inflammation

There was no statistically significant difference in baseline haematology parameters assessed for control and infected animals (Table 1). Similarly, no significant differences were observed in red blood cell parameters between control and infected animals throughout the experimental period. However, differences were observed in the white blood cell differential counts for control and infected animals (Table 1). The percentage of lymphocytes was significantly lower for infected animals at day 5 post surgery compared to controls (*P*=0.004). By day 28 post surgery, the proportion of lymphocytes was higher than baseline levels for both the control and infected group (*P*=0.032 and *P*=0.02, respectively). At day 5 post surgery, a significantly higher proportion of granulocytes was observed in blood from the infected group compared to controls, with levels returning to baseline range by day 10 post surgery (*P*<0.001; Table 1). Compared to baseline, total leucocyte numbers were lower at day 28 post surgery in infected animals (*P*=0.006), with a similar trend observed for control animals although this did not reach significance (*P*=0.07). Significant decreases were also observed in the total number and percentage of circulating monocytes in infected animals at day 10 post surgery, with levels remaining lower than baseline values at the end of the experimental period (Table 1).

**Table 1.**
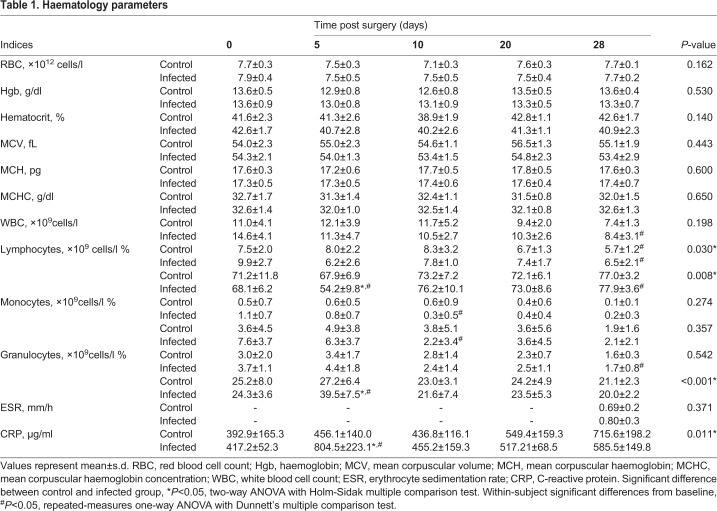
**Haematology parameters**

**Fig. 2.**
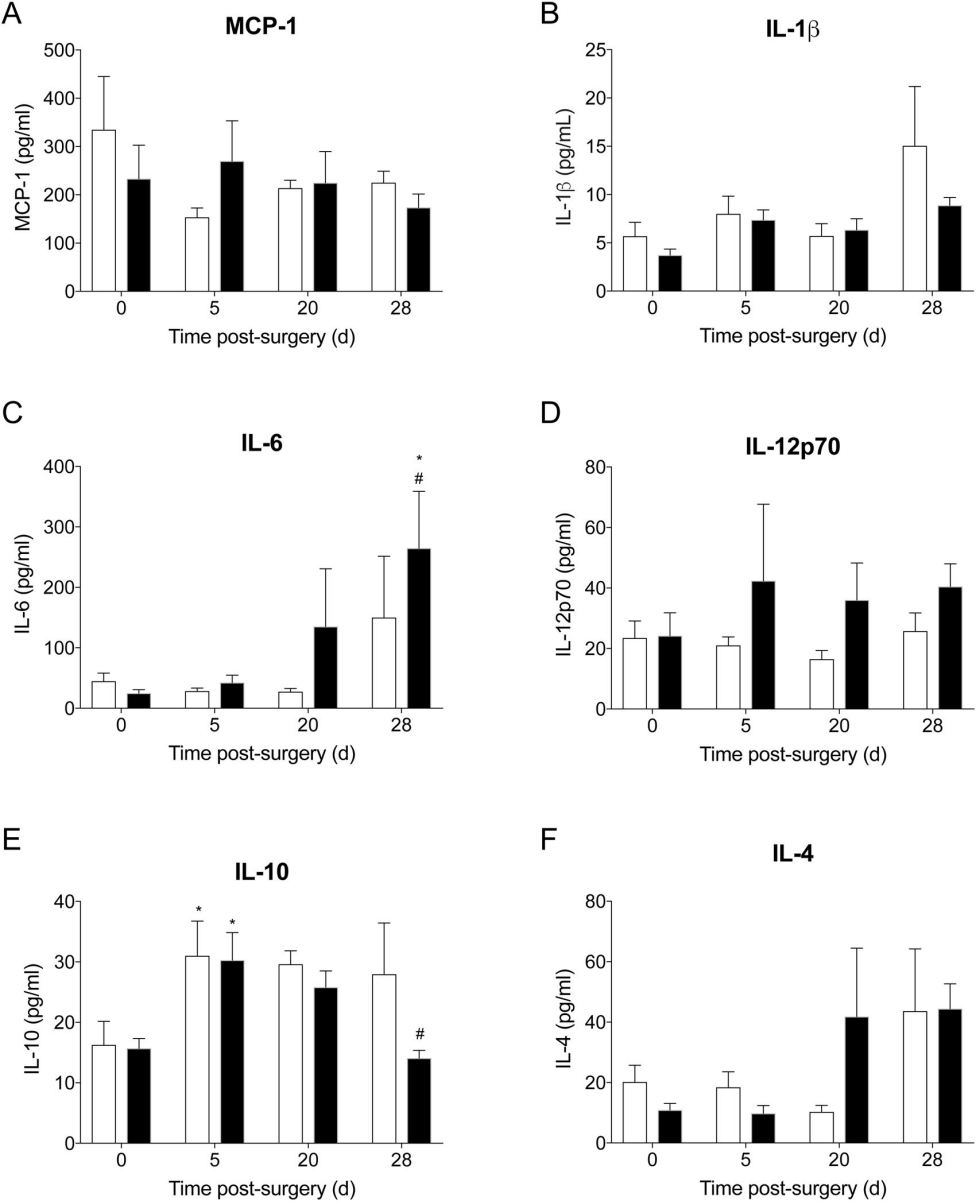
**Inflammatory chemokine and cytokines in plasma of control (*n*=5) and infected (*n*=10) animals at baseline, day 5, 20 and 28 post surgery.** Data show mean±s.e.m. *P<0.05 compared to baseline, ^#^*P*<0.05 compared to day 5 post surgery. Between-group comparisons, two-way ANOVA with Holm-Sidak multiple comparison test. Within-group comparisons, repeated-measures one-way ANOVA with Dunnett's multiple comparison test.

Erythrocyte sedimentation rate (ESR) was comparable for control and infected animals at day 28 post surgery (Table 1). Plasma C-reactive protein (CRP) concentrations in control animals remained unchanged over the 28-day period. In contrast, plasma CRP levels were increased in infected animals at day 5 post surgery, though concentrations returned to baseline levels by day 10 post surgery (*P*<0.001; Table 1). No significant differences were observed in plasma inflammatory chemokine and cytokine levels between control and infected animals across the experimental period (Fig. 2). TNF-α and IFN-γ levels remained at the assay limit of detection throughout the experimental period (data not shown). Plasma IL-10 levels were significantly higher at day 5 post surgery compared to baseline in both control and infected animals, returning to baseline levels by day 28 post surgery (*P*=0.033 and *P*=0.008, respectively; Fig. 2E). While there was a trend for increased IL-6 and IL-12p70 concentrations in plasma of infected animals at day 28 post surgery compared to controls, this did not reach statistical significance (Fig. 2C,D).

### Joint gross pathology

**Fig. 3.**
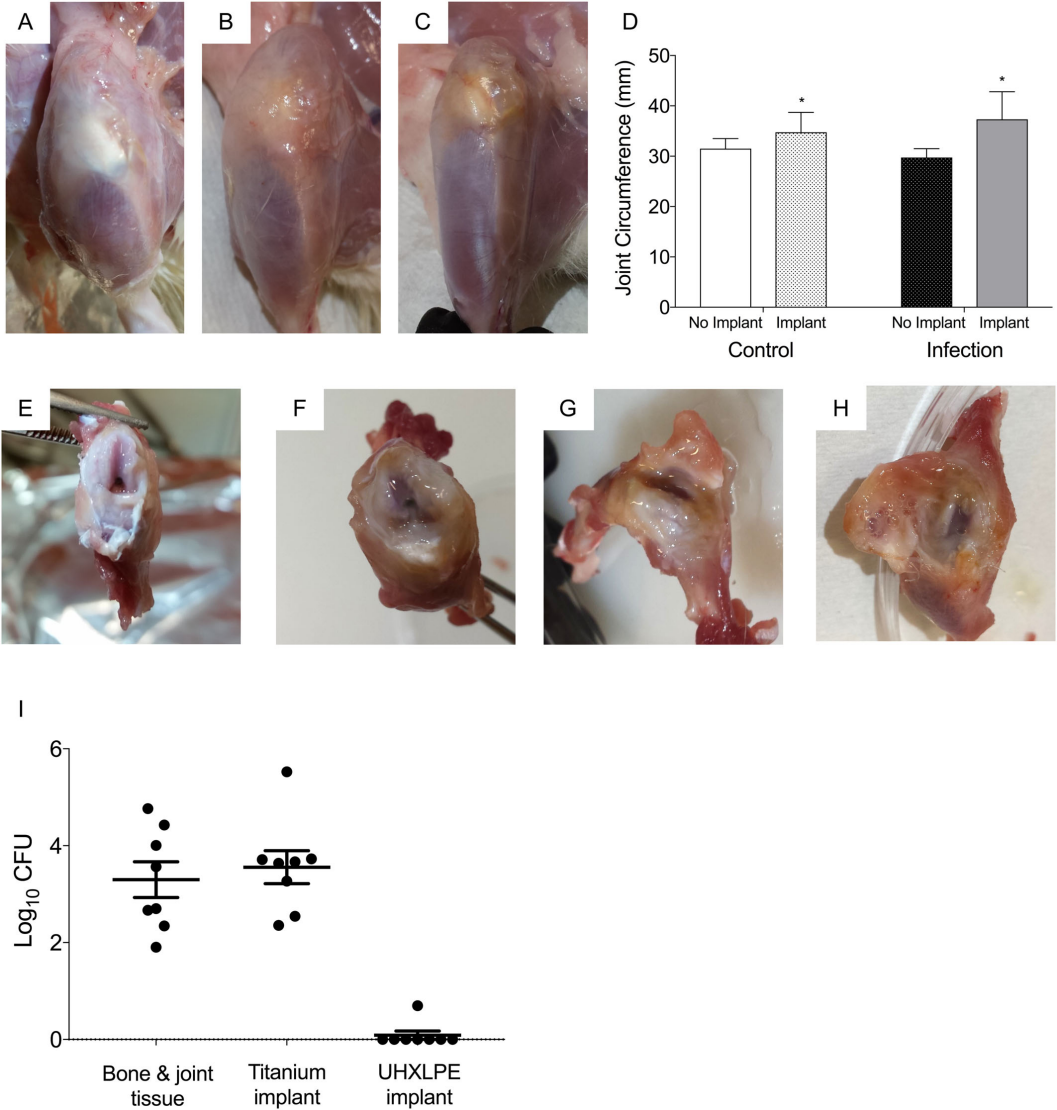
**Joint pathology and bacterial burden.** (A–C) Representative images of the operated hind limb of (A) control and (B,C) infected animals at day 28 post surgery**.** (D) Joint circumference of the non-operated (no implant) and operated (implant) limb of control (*n*=8) and infected animals (*n*=13) at day 28 post surgery. Data show mean±s.e.m. (not significant, *P*=0.12, Student's *t*-test; *P<0.05 compared to non-operated limb). (E–H) Representative images of dissected knee of (E) control and (F–H) infected animals at day 28 post surgery. (I) *S. aureus* was recovered from joint bone and soft tissue, and titanium implants of all animals in the infected group at day 28 post surgery. Data show mean±s.e.m.

Surgical incisions healed without complication in control and infected animals (Fig. S1B,C). Upon dissection, macroscopic examination of the operated knees of control animals at 28 days post surgery revealed mild soft tissue damage and clear synovial fluid (Fig. 3A,E), with increased joint circumference compared to the non-operated left knee (*P*=0.03; Fig. 3D). In contrast, mild-to-moderate soft tissue and articular cartilage damage was evident within joints of infected animals, often in combination with increased viscosity and amounts of synovial fluid (Fig. 3B,C,F–H). Joint circumferences of implanted knees were comparable between control and infected animals (*P*=0.12; Fig. 3D).

### Evaluation of implant stability

**Fig. 4.**
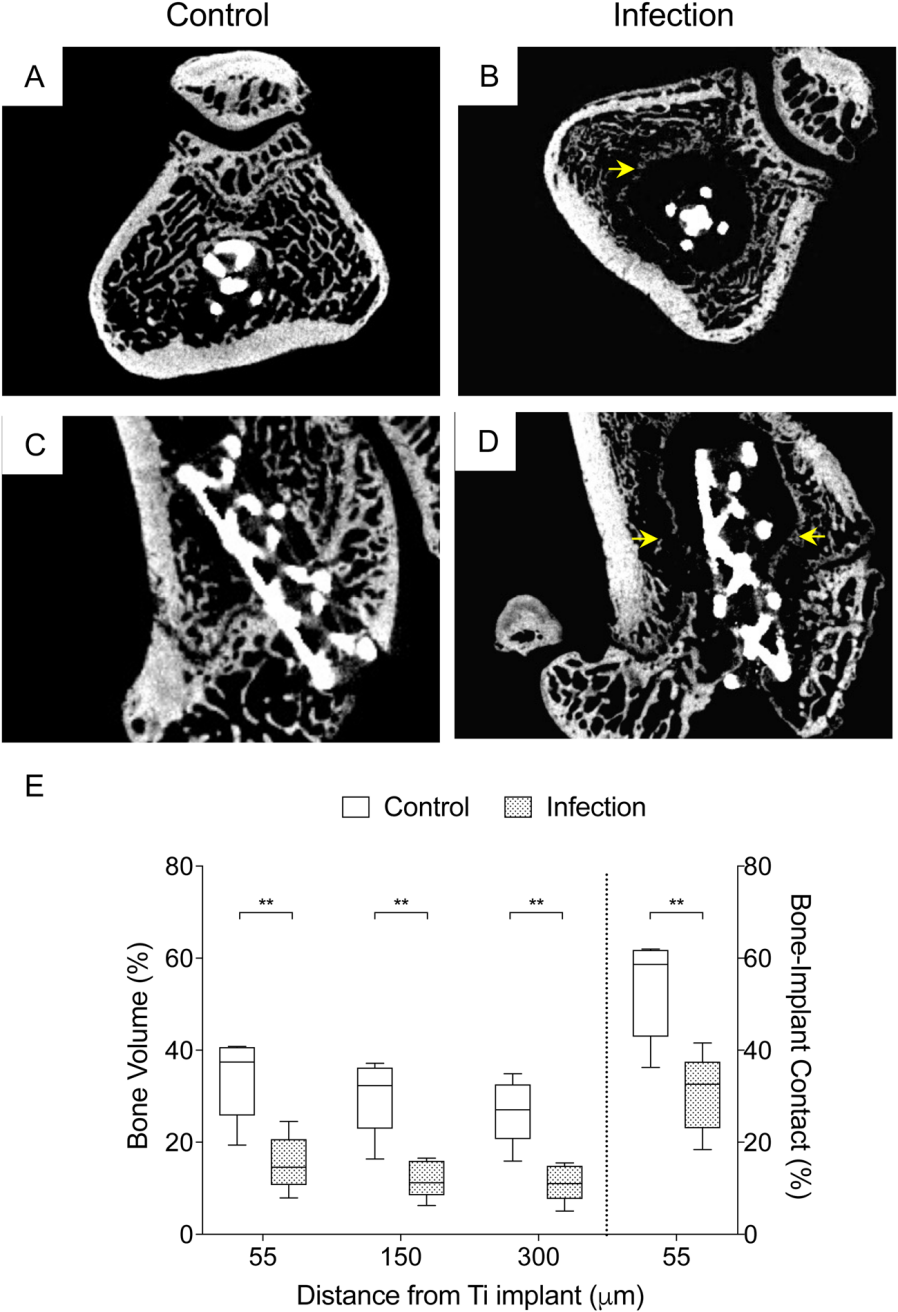
**Implant stability.** (A–D) Representative axial (A,B) and coronal (C,D) microCT scans of the distal femur in control and infected animals at day 28 post surgery. (E) The bone volume (BV) percentage within a 55, 150 and 300 µm distance from the implant surface, and bone-implant contact (BIC) percentage for the press-fit titanium implant was significantly lower in infected (*n*=5) compared to control animals (*n*=5) at day 28 post surgery. Data show median±i.q.r. **P<0.01, Mann–Whitney test.

Bone ingrowth was evident macroscopically within titanium implants excised from control animals at day 14 post surgery (Fig. S2). Micro-computed tomography (MicroCT) analysis of uninfected, control knees at day 7, 14 and 28 post surgery confirmed that both the titanium and UHXLPE implants were stable and well-positioned (Fig. S2). Increases in bone volume (BV) surrounding the press-fit titanium implant was evident between day 7 and 14 post surgery (*P*=0.041) in control animals, followed by a decrease from day 14 to 28 post surgery (*P*<0.001; Figs S2 and S3). Similarly, bone-implant contact (BIC) for the titanium implant increased in the first 2 weeks (*P*=0.002), then decreased slightly by day 28 post surgery, though BIC remained significantly higher than 7 days after surgery (63.7% versus 72%, *P*=0.02; Figs S2 and S3). Bone parameters were not quantitatively assessed for the tibial UHXLPE implant since the bone cement mantle it was constrained within typically filled >80% of the tibial metaphyseal area. However, new bone formation and bone-cement contact was evident from histological and microCT images, with no tibial implant loosening observed in either the control or infected animals at day 28 post surgery (Fig. S2). Compared to control animals, BV and BIC were significantly lower surrounding the femoral titanium implant in infected animals at day 28 post surgery, with evidence of peri-implant osteolysis and bone remodelling in all animals assessed (Fig. 4).

### Microbiology

Sterility of the surgical site in control animals was confirmed by the absence of bacteria in cultures from tissue homogenates of the spleen, draining lymph nodes, femur, tibia, patella and soft tissue of the operated knee at day 28 post surgery (Table 2). Similarly, no bacteria were recovered from the titanium or UHXLPE implants removed from control animals.

**Table 2.**
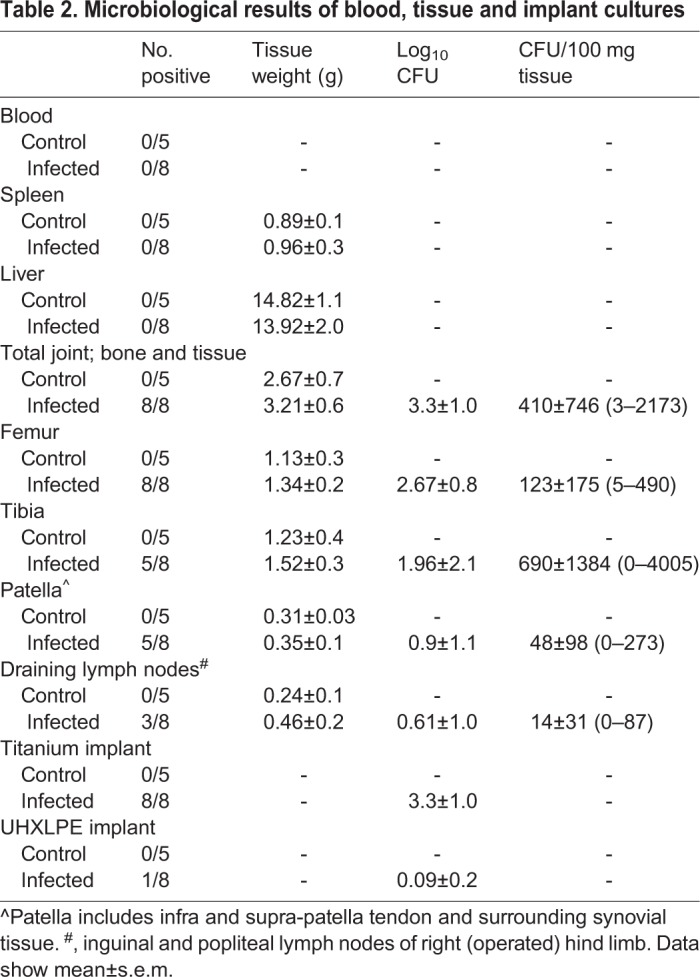
**Microbiological results of blood, tissue and implant cultures**

Blood cultures from infected animals remained negative throughout the experimental period and at end-point analysis. Similarly, no bacteria were recovered from spleen or liver of animals in the infected group, with tissue weights comparable to control animals (Table 2). Bone and joint tissue weights of dissected knees were also comparable between control and infected animals. At day 28 post surgery, *S. aureus* was cultured from joint tissue and implants of all animals that had received pre-seeded titanium implants. Bacterial loads were highest for titanium implants (range, 2.5×10^2^–3.2×10^5^ CFU) and femur (range, 55–8.5×10^3^ CFU). Bacteria were recovered from tibia, patella and surrounding capsular tissue in five of eight infected animals, with considerable variability in bacterial numbers in these tissues between animals (Table 2). *S. aureus* was also cultured from draining lymph nodes of the implanted knees of three of eight animals at day 28 post surgery (mean, 98 CFU).

### Joint inflammation and histopathology

**Fig. 5.**
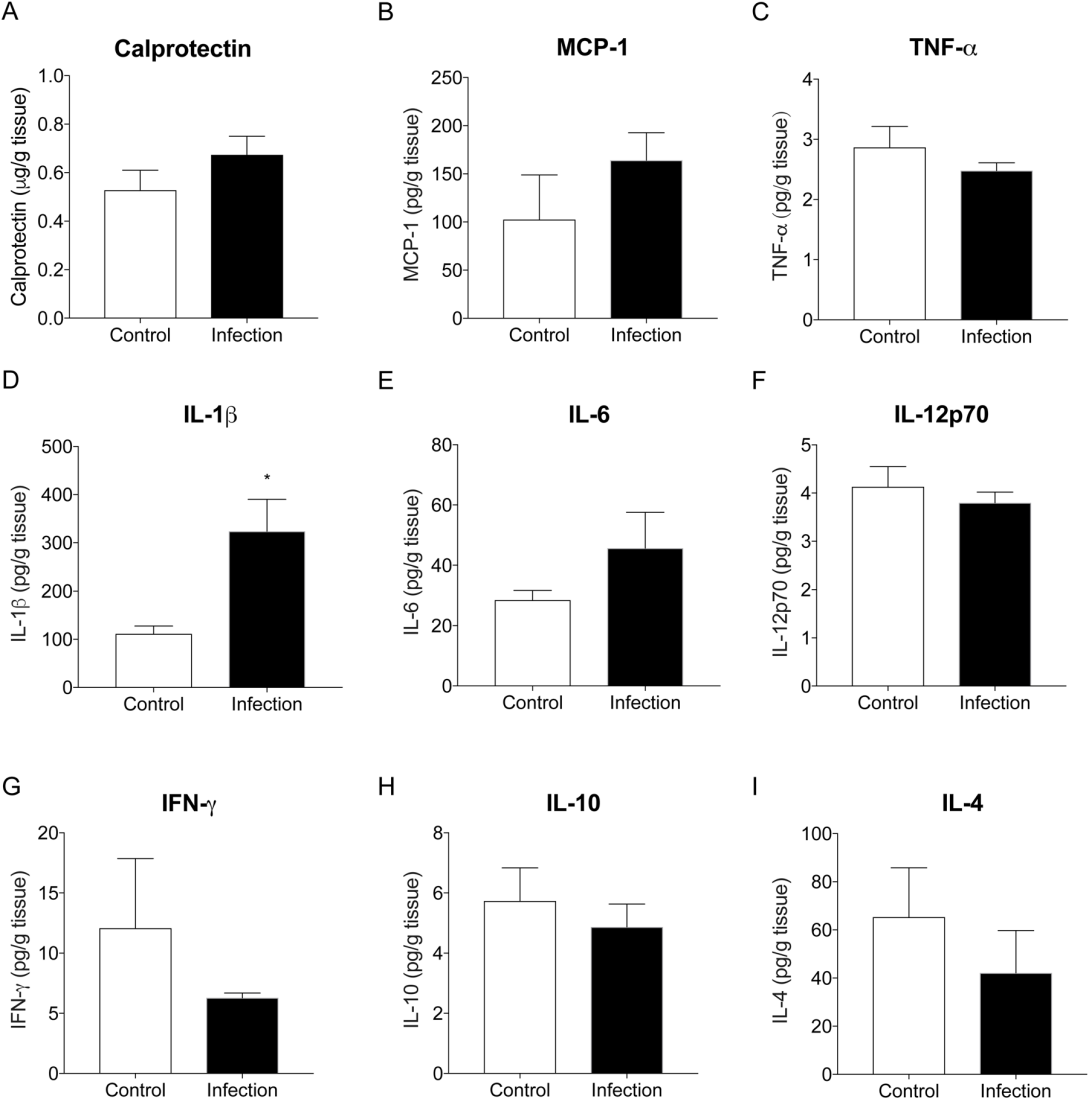
**Inflammatory chemokines and cytokines in joint tissue of implanted knees from control (*n*=5) and infected (*n*=10) animals at day 28 post surgery.** Data show mean±s.e.m. *P<0.05, Mann–Whitney test.

Compared to control animals, IL-1β was significantly higher in joint tissue of infected animals at sacrifice (*P*=0.013; Fig. 5D). While differences were not statistically significant, there was a trend for increased levels of calprotectin, MCP-1 and IL-6 in joints of infected compared to control animals (Fig. 5A,B,E). In contrast, levels of TNF-α, IFN-γ, IL-10 and IL-4 tended to be lower in joint tissue from infected compared to control animals, though these differences were not statistically significant (ns, Fig. 5).

**Fig. 6.**
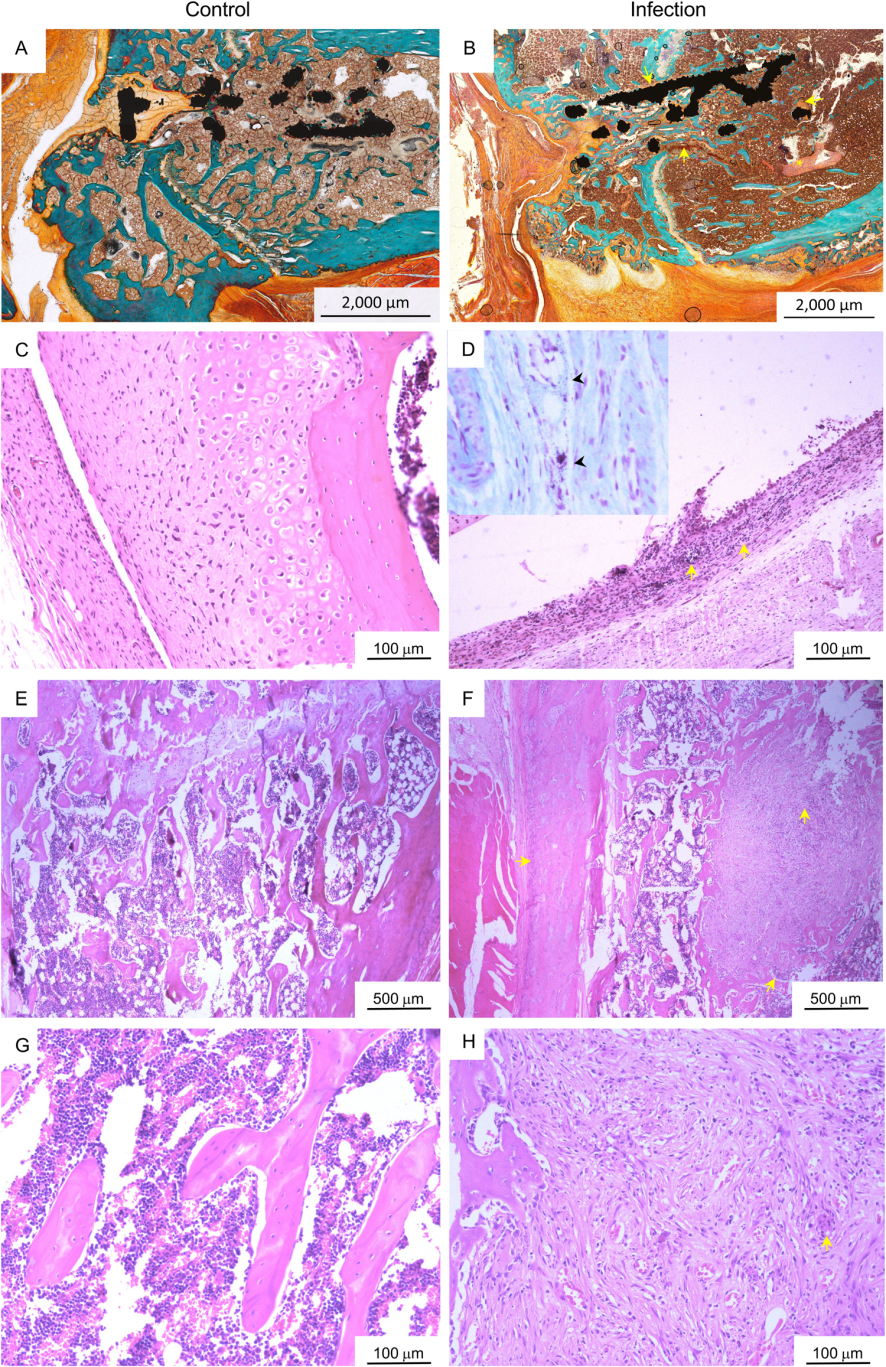
**Joint histopathology.** (A,B) Representative images of Goldner Trichrome-stained, resin-embedded femur with titanium implant from (A) control and (B) infected animals at day 28 post surgery, where mineralised bone matrix, erythrocytes and non-mineralised osteoid (new bone) are stained green, orange and red, respectively. Focal areas of fibrosis (arrows) and necrosis (asterisk) were evident in peri-implant tissue of the infected animals. (C–H) Representative histological images of implanted knees from control (C,E,G) and infected (D,F,H) animals at day 28 post surgery stained with Haematoxylin and Eosin**.** Compared to control animals (C), synovial hyperplasia and inflammatory cell infiltration (arrows) was evident in joint capsular tissue of the implanted knee of infected animals (D) at day 28 post surgery, with occasional cocci observed with Gram-Twort staining (inset, arrowheads). Loss of normal bone architecture, peri-implant fibrosis and inflammatory cell infiltration (arrows) were observed adjacent to the titanium implant and in the periosteum within the femur of infected animals (F,H) compared to control animals (E,G).

Joint histopathology was consistent with gross pathology, inflammatory cytokine and microCT findings. Direct bone contact with the titanium implant was evident in the femur of control animals, with new, non-mineralised bone formation at the proximal and distal zones of the implant (Fig. 6A). There was no evidence of peri-implant inflammation or osteolysis in sections from control animals at day 28 post surgery (Fig. 6E,G). Similarly, no inflammatory changes were observed in synovial tissue from knees of control animals (Fig. 6C). In contrast, synovial hyperplasia and infiltration of inflammatory cells into joint capsule tissue was observed in infected animals (Fig. 6D). Small foci of gram-positive cocci were also occasionally observed within joint synovial tissue of infected animals (Fig. 6D, inset). Destruction of normal bone architecture, woven bone formation and fibrosis was evident in peri-implant tissue of the femur, with increased inflammatory cell infiltration and small focal areas of lysis in animals infected with *S. aureus* (Fig. 6B,F,H). Compared to control animals, no obvious histopathological differences were observed within the tibia of infected animals.

## DISCUSSION

Implant failure due to bacterial infection continues to be a devastating and costly complication associated with TKA, with treatment typically involving multiple surgical procedures and prolonged courses of antibiotic therapy (Peel et al., 2013; Tande and Patel, 2014). MSSA causes approximately 45% of PJI cases, and is 2.5 times more likely to be associated with a PJI than MRSA (Guo et al., 2017; Tande and Patel, 2014). Despite apparent antibiotic sensitivity *in vitro*, the ability of MSSA strains to form biofilms on orthopaedic implants *in vivo* continues to pose a significant challenge for treatment of delayed- and late-onset PJI. Identifying alternate strategies for treating PJI is currently a priority area of research, and small animal models will continue to play a key role in translation of new therapeutic approaches (Schwarz et al., 2019). While several rat models of implant-associated *S. aureus* infection have been described, clinical representation of modern TKA in terms of the combination of orthopaedic materials used has been limited (Lin et al., 2017; Reizner et al., 2014; Schindeler et al., 2018; Stadlinger et al., 2013). Previously described models have used intramedullary insertion of stainless steel implants into the distal femur or the proximal tibia (Edelstein et al., 2017; Lucke et al., 2003), or extra-articular insertion of screws or pins into bone (Stadlinger et al., 2013). We describe the surgical technique for a novel rat model of knee implant surgery based on materials currently used in TKA. Further, using a previously characterised clinical MSSA strain to colonise the femoral titanium implant prior to surgery, we successfully progressed our uncomplicated surgical model to one representative of delayed-onset PJI.

Additive manufacturing technology, or 3D printing, is increasingly being applied to orthopaedics as a result of its capacity to blend different materials such as titanium into diverse pore sizes and thicknesses so as to mimic the porosity and stiffness of bone (Bravin and Dietz, 2018). Newer generation TKA implants comprise 3D-printed highly porous titanium-coated components that exhibit a high degree of osseointegration following cementless fixation (Bravin and Dietz, 2018). UHXLPE is commonly used for covering articulating surfaces due to its favourable wear rate (Dion et al., 2015; Le et al., 2014). Recently, Carli et al. (Carli et al., 2018) described a mouse model of implant-related osteomyelitis based on a press-fit titanium tibial implant comprised of an articular baseplate and intramedullary stem. Using intra-articular inoculation of *S. aureus* at the time of surgery, the authors report the establishment of high-level, purulent infection with elevated systemic inflammatory markers (Carli et al., 2018). While this model offers the advantage of an implant with a weight-bearing surface, it may not accurately represent the clinical features of delayed-onset, low-level PJI for which blood cultures remain negative and systemic inflammatory markers are often within normal ranges (Beam and Osmon, 2018). To our knowledge, only one study has used a combination of metal and high-density polyethylene press-fit femoral and tibial implants for development of a rat model of PJI (Søe et al., 2013). Using non-constrained knee prostheses, Søe et al. (2013) demonstrated establishment of osteomyelitis in rats following direct seeding of >10^3^ CFU *S. aureus* into both the femoral and tibial defects at the time of knee surgery. Semi-quantitative methods were subsequently used to infer implant stability and bacterial persistence in this non-constrained model (Søe et al., 2013). Notably, implant stability and long-term success of TKA is dependent on component fixation that can either be cemented (constrained) or cementless (non-constrained) (Dalury, 2016; Vertullo et al., 2018). Antibiotic-laden bone cement was introduced in the 1970s for infection prophylaxis in arthroplasty, with gentamicin one of the most commonly incorporated antibiotics (Hinarejos et al., 2015). The distinction of our model is the use of a hybrid fixation technique, making it more akin to the materials and techniques currently used in TKA (Dion et al., 2015; Hinarejos et al., 2015; Le et al., 2014; Lin et al., 2017) and the establishment and persistence of a low-level PJI that is localised to the operated knee.

In the absence of infection, animals in the current study recovered without complication and returned to full weight-bearing within the first week of knee implant surgery. Although plasma CRP levels were higher at day 28 post surgery in control animals than at baseline, these levels remained within reported normal ranges for CRP in rats (de Beer et al., 1982). Further, haematology parameters, including leucocyte number, had returned to baseline levels by day 28 post surgery, with no additional clinical, pathological or histological evidence of inflammation in the operated limb of control animals. Importantly, stability of both the femoral and tibial implants, and bone ingrowth around the press-fit porous titanium implant and cement mantle surrounding the UHXLPE implant was evident within 4 weeks of surgery in the absence of infection in control animals.

In the current study, pre-seeding of femoral titanium implants with MSSA was sufficient to establish a persistent, non-lethal and localised infection in 100% of animals. While some systemic inflammatory markers (CRP, % PMN) were significantly higher in infected animals compared to controls in the first week following surgery, levels returned and were comparable to controls for the remainder of the 4-week experimental period. This is consistent with previous animal models of implant-associated osteomyelitis (Carli et al., 2018; Lucke et al., 2003; Rissing et al., 1985) and the clinical features of delayed-onset PJI, where serum biomarkers have proven unreliable for diagnosis (Beam and Osmon, 2018). Similar to the clinical manifestations of delayed-onset PJI (Beam and Osmon, 2018), overt signs of persistent infection in animals in the current study included a sustained reduction in post-surgical body weight and delayed return to full weight-bearing on the operated limb.

Localisation of MSSA infection to the implanted knee was confirmed in the current study by quantitative microbiological, immunological and radiographic changes. Culture of peri-prosthetic and sonicate fluid remains the gold standard for diagnostic confirmation of PJI (Beam and Osmon, 2018; Schwarz et al., 2019; Tan et al., 2018). Despite the use of antibiotic-laden bone cement, bacteria were recovered from all excised titanium implants and peri-prosthetic tissue of the femur, and from tibia and patella, demonstrating spread of MSSA to adjacent tissues within the joint. Although there was inter-individual variability in the distribution of bacteria within joint tissue, the total number of MSSA recovered from implanted joints at 4 weeks post surgery was consistent (∼2×10^3^ CFU). Bone remodelling, metaphyseal osteolysis, fibrosis and inflammatory cell infiltration was apparent surrounding the press-fit titanium implant of infected animals at day 28 post surgery, with a 2.4-fold decrease in peri-implant BV compared to uninfected control animals. Consistent with this, levels of IL-1β, which activates osteoclasts to increase bone resorption (Nguyen et al., 1991), were significantly increased in the implanted knee of infected compared to control animals.

Synovial rather than serum inflammatory markers consistently demonstrate greater diagnostic sensitivity for PJI. Measurement of synovial IL-6, CRP, alpha-defensin, leukocyte esterase and calprotectin have been shown to improve diagnostic accuracy in the workup for PJI (Beam and Osmon, 2018; Wouthuyzen-Bakker et al., 2018; Xie et al., 2017). Nevertheless, published cut-off points for these biomarkers vary widely for PJI diagnosis. The reported cut-off values for synovial fluid IL-6 and CRP, for example, range from 359.3 to 30,750 ng/l, and 3.65 to 12.2 mg/l, respectively, in patients with PJI following knee or hip arthroplasty (Gallo et al., 2018). Synovial fluid was not measured in the current study. However, in addition to elevated IL-1β, levels of calprotectin, IL-6 and MCP-1 tended to be higher in peri-implant tissue of infected animals compared to controls. Calprotectin is an antimicrobial protein that abundantly presents in the cytoplasm of neutrophils and is released upon an encounter with pathogens. While neutrophils predominate in the acute response to infection, their numbers at the site of low-grade or chronic infection tend to be lower, with lymphocytes and monocytes also contributing to the leukocyte milieu in PJI (Josse et al., 2019). IL-6 is produced by stromal and activated immune cells at sites of inflammation or infection and plays a key role in regulating not only early neutrophil infiltration, but also, together with monocyte chemoattractant protein 1 (MCP-1), the mononuclear cell shift that occurs during prolonged inflammation or chronic infection (Gabay, 2006). The minor elevations observed in joint tissue inflammatory markers were consistent with the mild-to-moderate mixed neutrophil, monocyte and lymphocyte infiltration into synovial and peri-implant tissue in knees from infected animals.

The major limitations of this study were the non-tribological nature of the implants, the non-physiological method used for inoculation and the relatively high numbers of *S. aureus* pre-seeded to the titanium implants. The inoculation dose used in our study is consistent with those reported for other rat PJI models using different *S. aureus* strains (Reizner et al., 2014). Direct inoculation of bacteria into the joint at the time of surgery was not used since planktonic bacteria are more readily cleared by host immune responses or potentially disseminate to surrounding tissues or distant sites via haematogenous spread, thus increasing variability in the infection site and the inoculating dose within the joint, and the capacity for establishing infection (Arciola et al., 2018). Pre-seeding of the titanium implants with ∼2×10^4^ CFU provided a reliable method for establishing a low-level, persistent and localised infection in rats that is detectable 4 weeks after inoculation. We emphasise that our purpose was to create a reliable experimental model that reflects the key characteristics of delayed-onset PJI, rather than its pathogenesis, per se. Importantly, our approach consistently achieved the clinical features of delayed-onset PJI, where osteomyelitis was initiated in the bone marrow adjacent to the implant causing bone remodelling and local inflammatory changes in the absence of systemic manifestations or clinically overt signs of infection. Similar to the clinical scenario, no single biomarker or test could reliably be used in lieu of microbiological culture to confirm PJI in our experimental model. An additional limitation of the study was that scanning electron microscopy (SEM) was not used to visualise the structure of mature biofilms on explanted titanium scaffolds at the study end as all samples were used for bacterial quantification. Nonetheless, given bacterial loads were consistently highest from titanium implant sonicates, it is likely that the localised, persistent infection observed at day 28 post surgery was associated with biofilm formation on the implant surface. The pull-out strength of implants was also not assessed in the current study. Rather, implants were carefully excised to improve accuracy of bacterial load determination with osseointegration and implant stability assessed by microCT analysis of peri-implant BV and bone-implant contact.

In summary, we describe a surgical technique for knee implant surgery in rats that is simple, reproducible, economical and recreates the peri-prosthetic space akin to modern TKA. Further, key diagnostic features of delayed-onset PJI (Beam and Osmon, 2018) were demonstrated, with impaired return of joint function, sustained weight loss after surgery, modest increases in inflammatory markers within peri-implant tissue, evidence of peri-implant bone remodelling, and positive culture from joint tissues and implant sonicates. This model will serve as a useful and clinically relevant tool for facilitating bench-to-bedside translation of new treatment approaches for delayed-onset PJI caused by *S. aureus* biofilms.

## MATERIALS AND METHODS

### Bacterial biofilms

A previously described, a clinical isolate of *S. aureus*, ORI16_C02N, was used in the current study (Morris et al., 2018). ORI16_C02N was isolated from a patient who had presented with septic arthritis 3 years post TKA. This strain has sensitivity to gentamicin, cefazolin and flucloxacillin. ORI16_C02N is negative for panton-valentine leucocidin (pvl) and the plasmids, pUB110 and pT181, positive for collagen adhesion (cna) and serine-aspartate repeat-containing protein E (sdrE), and belongs to clonal complex (CC) 78.

In preliminary studies, BV SEM demonstrated the establishment of mature ORI16_CO2N biofilms on the titanium implant surface within 24 h of culture (Fig. S4). To more closely represent the early stages of biofilm formation (adherence and colonisation), sterile titanium implants were suspended in log phase cultures of ORI16_C02N (1.8×10^4^ CFU, range 1.3–2.9×10^4^ CFU) for 12 h at 37°C, with a media change performed after 2 h to remove non-adherent bacteria. Prior to implantation in rat femurs, biofilm-coated titanium implants were rinsed twice in saline. Confirmation of mean bacterial density within 12 h biofilms established on custom titanium implants (*n*=5) was determined to be 1.2×10^6^ CFU (range, 8.9×10^5^–1.9×10^6^ CFU) using sonication and enumeration of colonies on tryptic soy agar (TSA) (Morris et al., 2018). Complete disruption of the biofilm was confirmed with SEM (Fig. S4).

### Animals

Twenty-week-old male Sprague-Dawley rats (350–450 g) were used. Animals were individually caged, fed a standard pellet diet and provided water *ad libitum*. At commencement of the 7-day acclimation period prior to surgery, animals were randomised to control (*n*=12), or *S. aureus*-infected (*n*=13) groups. Animals in the infected group received titanium implants that had been pre-coated with an *S. aureus* biofilm (described below). Control animals received sterile titanium implants. Clinical signs including body weight, temperature and weight-bearing activity were monitored daily throughout the experimental period. Animals were sacrificed at 28 days post surgery with an overdose of pentobarbital (100 mg/kg) for gross pathology, haematology, microbiology, inflammatory, microCT and histological evaluation. All animal experimental procedures were approved by the Institutional Animal Ethics Committee (A2326).

### Implants

3D-printed porous titanium implants were produced from Ti-6Al-4V, the alloy used in human components, as previously described (Mazur et al., 2016). The porous architecture was based on a cylindrical scaffold measuring 5 mm×1.6 mm, with a strut width of 205 µm and 70% porosity. Custom cylindrical UHXLPE implants (5 mm×1.6 mm) were kindly produced and provided by Enztec (Christchurch, New Zealand).

### Surgical technique

All surgeries were performed within a sterile surgical field, using aseptic techniques, sterile instruments, gowns, gloves and drapes. Knee implant surgery was performed on rats under general anaesthetic with 5% isoflurane (in 100% oxygen) during the induction phase and 2.5% isoflurane during surgery, with animals breathing spontaneously (Fig. S5). Hair on the right hind limb (ankle to abdomen) was clipped using sterile scissors, then completely removed using hair removal cream (Veet^®^). The shaved area was cleaned with chlorhexidine wash then swabbed liberally with 70% ethanol. The right hind foot was swabbed liberally with alcoholic povidone-iodine solution (10% w/v) using a sterile gauze and air-dried. Once dry, 3M™ Tegaderm™ was applied around the foot. The shaved, right hind limb was swabbed liberally with povidone-iodine and air-dried. A small fenestration (2 cm×1.5 cm) was made in a large sterile drape (120 cm×120 cm) through which the right knee was positioned so as to maintain a sterile surgical field. Sterile IV3000^®^ was applied over the flexed knee and fixed to the surrounding drape.

The knee was opened with a medial parapatellar incision (12 mm) and the patella dislocated laterally to expose the femoral condyles and tibial plateau. A Microdremel and sterile titanium carbide drill bit (2 mm) was used to create a defect in the proximal tibia. The UHXLPE implant was seated in a small mantle of gentamicin-laden bone cement (Heraeus Palacos^®^ R+G, Zimmer Biomet, Sydney, Australia) which was mixed according to ratios outlined in the manufacturer’s guidelines. Bone cement was deployed into the tibial defect using a sterile, 3 ml syringe (Terumo, Sydney, Australia) and the implant seated by gently tapping into place using sterile forceps. The Microdremel and a second sterile titanium carbide drill bit (1.6 mm) were used to create a defect between the distal medial and lateral femoral condyles. Using forceps, the titanium implant was press-fit into the femoral defect using gentle tapping. Following implantation, the patella was repositioned and polydioxanone (PDS II) 5-0 (Ethicon, New Jersey, USA) sutures were used to close the capsule. Skin was closed with monocryl 5-0 (Ethicon) using a continuous subcuticular technique and the surgical site swabbed with povidone-iodine solution, then sprayed with OpSite™. Immediately after skin closure and prior to recovery from anaesthesia, animals received pre-emptive analgesic consisting of a 0.05 mg/kg subcutaneous injection of buprenorphine (Temgesic^®^) in a 1 ml bolus of saline. Two additional doses of buprenorphine (0.05 mg/kg) were administered at 6 and 12 h post surgery, with analgesic administered 8 to 12 times hourly thereafter, according to pain scores of individual animals. Clinical signs including body weight, temperature and weight-bearing activity were monitored daily throughout the experimental period.

### Haematology and inflammatory assessments

Blood was collected under anaesthesia via the lateral tail vein during the experimental period or via terminal cardiac puncture at day 28 post surgery. Complete blood cell examination (CBE) was carried out using an automated ACT Diff analyser (Beckman Coulter, Brea CA, USA). Blood samples were centrifuged and plasma collected and stored at −80°C until further analysis. CRP was measured in plasma using a Rat CRP ELISA (BD Biosciences, North Ryde, Australia) according to the manufacturer's protocols. Calprotectin was measured in joint tissue homogenates using a Rat Calprotectin ELISA (Cusabio, Houston, TX, USA) according to the manufacturer’s protocols. Inflammatory chemokines and cytokines (MCP-1, GRO/KC, MIP-2, TNF-α, IL-1β, IL-6, IL-12p70, IFN-γ, IL-4, IL-10) were measured in plasma and joint tissue homogenates using Milliplex^®^ Rat Cytokine/Chemokine Magnetic Bead Panel (Abacus ALS, Meadowbrook, Queensland) in combination with the Magpix^®^ analyser (Luminex Corporation, Austin, Texas, USA). Assays were carried out according to the manufacturer's instructions with samples measured in duplicate. Detection ranges for analytes were: 29.3–120,000 pg/ml for MCP-1; 14.6–60,000 pg/ml for IFN-γ and GRO/KC; 24.4–100,000 pg/ml for MIP-2; 2.4–10,000 pg/ml for IL-1β and TNF-α; 73.2–300,000 pg/ml for IL-6; 12.2–50,000 pg/ml for IL-12p70, 4.9–20,000 pg/ml for IL-4; and 7.3–30,000 pg/ml for IL-10. Assay sensitivities [minimum detectable concentration (pg/ml), intra-assay precision (% CV) and inter-assay precision (% CV, *n*=11 assays)] for each analyte were: MCP-1: 9.0, 2.3, 9.2; GRO/KC: 19.7, 5.4, 7.7; MIP-2: 11.3, 2.9, 7.7; TNF-α: 1.9, 2.7, 10.8; IL-1β: 2.8, 3.6, 11.3; IL-6: 30.7, 2.3, 12.7; IL-12p70: 3.3, 2.2, 7.8; IFN-γ: 6.2, 2.7, 12.4; IL-4: 3.1, 3.1, 10.7; and IL-10: 2.7, 3.8, 9.

### Microbiological analyses

Bacterial loads were determined in blood, tissues and implants from animals (control, *n*=5; infected, *n*=8) at day 28 post surgery. Briefly, liver, spleen, popliteal and inguinal (draining) lymph nodes of the operated (right leg) and non-operated (left leg) limb were dissected aseptically. The operated limb was also removed at the hip and soft tissue was removed from the femur and tibia and bone cutters used to cut the bone into small (<5 mm) pieces. Titanium and UHXLPE implants were removed and sonicated as described previously (Morris et al., 2018). The patella, associated tendons and capsular tissue were processed separately from femur and tibia. Tissues were homogenised using sterile stainless-steel beads (0.9–2.0 mm blend) and Bullet Blender (NextAdvance, Troy, NY, USA). Blood and tissue homogenates were serially diluted and cultured on TSA and mannitol salt agar (MSA) overnight to enumerate bacteria.

### Radiographic evaluation

Anterior–posterior (AP) and lateral x-ray images were taken of hind limbs of animals (*n*=2) on day 7 post surgery to confirm implant positioning (55–60kVp, 200 mA, 32 m s−1, 6.3 mAs; Shimadzu general unit and digital detector plate, Canon CXDi-50G, Kyoto, Japan).

### Micro-computerised tomography (µCT) scans

After sacrifice, hind limbs were removed from animals, with muscle and soft tissue dissected from bone leaving the knee capsule intact (control, *n*=5; infected, *n*=5). Specimens were fixed in 4% paraformaldehyde (48 h, 4°C) prior to performing *ex vivo* microCT [Inveon PET-CT, Siemens, Bayswater, Australia; 16.6 µm voxel size, 80 E(kVp), 200 µA, 0.36° rotation step]. Scan images were reconstructed and bone parameters surrounding the press-fit, femoral titanium implant were assessed using Materialise Mimics Innovation Suite *v*20 (Materialise, Leuven, Belgium) with scan and reconstruction parameters identical for all specimens.

Global thresholds were used to distinguish bone, soft tissue and the titanium implant (bone, 1020–4142; titanium implant, 4142–20546). Thresholds were based on visual inspection and were kept constant for all scans. A one-voxel border surrounding the implant was excluded to account for beam-hardening artefacts due to the metallic implant. Bone volume (%) was determined within a cylindrical volume of interest (VOI) surrounding the titanium implant using a series of mask dilations (55, 150 and 300 µm) from the implant surface, then Boolean subtraction was used to determine the intersect between bone within each sub-volume. Bone-implant contact (BIC, %) was calculated as a percent ratio of the bone-implant intersect area at a distance of 55 µm from the implant surface and the total surface area of the titanium implant.

### Histology

Following µCT scanning, representative knees were processed for resin-embedded (control *n*=1, infected *n*=1) or routine histology (control *n*=3, infected *n*=3). Briefly, hind limbs were dissected at the knee to separate the femur and tibia without damaging the condylar surfaces. For resin histology, bones were dehydrated using an ethanol series with increasing concentration. Following dehydration, infiltration was conducted using a mixture of ethanol and Technovit 9100 resin (Heraeus Kuzler, Hanau, Germany), with an increasing ratio of resin over a period of 3 weeks. Specimens were embedded using a UV embedding system and the polymerised specimen block was longitudinally or transversely sectioned at each implant centre using an EXAKT diamond cutting system (Kulzer Exakt 300 CP). Blocks were attached to slides using an adhesive press system and final slides were ground to a thickness ranging from the initial 200 µm to 48±5 µm using an Exakt grinding system (Kulzer Exakt 400 CS). Goldner's Trichrome staining was performed prior to mounting the sample and the final slide images were scanned with a microscope (Axio M2 Imager, Carl Zeiss, Gottingen, Germany). For routine histology, intact knee joints were decalcified in 14% EDTA, then bisected in an axial plane on the lateral edge of the implant to enable sectioning of peri-implant tissue, avoiding excessive tissue damage that would result from removal of the titanium implant. Samples were processed and paraffin-embedded sections (4 µm) stained with Haematoxylin and Eosin (H&E).

### Statistics

*A priori* power analysis was conducted using the G-power^3^ program to determine appropriate sample size to reduce Type 1 errors (CFU in joint tissue at day 28 post surgery; *n*=8). Statistical analyses were performed using GraphPad Prism for Mac software (version 7). Data normality was assessed using Shapiro-Wilks test, with Levene's test used to determine equality of variances. Independent samples *t*-tests were used for between-groups comparison for normally distributed data. Changes in haematology parameters for control and infected animals were compared using two-way repeated measures ANOVA with Holm-Sidak post-hoc analysis. Within-group differences were analysed with paired samples *t*-tests or, where appropriate, repeated measures ANOVA with Dunnett's post-hoc analysis. Non-normally distributed data were compared using a Mann–Whitney *U*-test or Kruskal–Wallis test with Dunn's post-hoc analysis. MILLIPLEX Analyst 5.1 software (Luminex Corporation, Austin, Texas, USA) was used to determine cytokine and chemokine concentrations with a 5-parametric logistic weighted curve fit. Results are expressed as mean±standard deviation (s.d.), with significance set at *P*<0.05.

## Supplementary Material

Supplementary information
