# Supplementary material for: Experimental model of peri-prosthetic infection of the knee caused by Staphylococcus aureus using biomaterials representative of modern TKA
Source: Biol Open. 2019 Sep 15;8(9):bio045203. doi: 10.1242/bio.045203 (PMC6777360; doi:10.1242/bio.045203)
Supplement: Supplementary information [file biolopen-8-045203-s1.pdf]

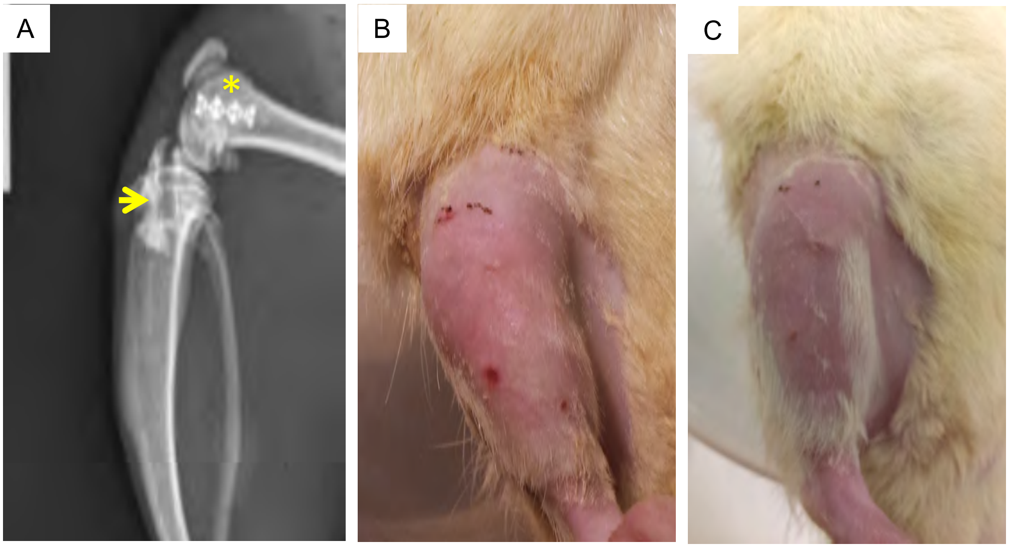

Figure S1. A) Representative radiograph of operated knee of a control animal at day 14 post-surgery showing placement of the femoral press-fit titanium implant (asterisk) and cemented tibial UHXLPE implant (arrowhead). Representative images of the surgical incision site of infection animals at B) day 5 and C) day 10 post-surgery.

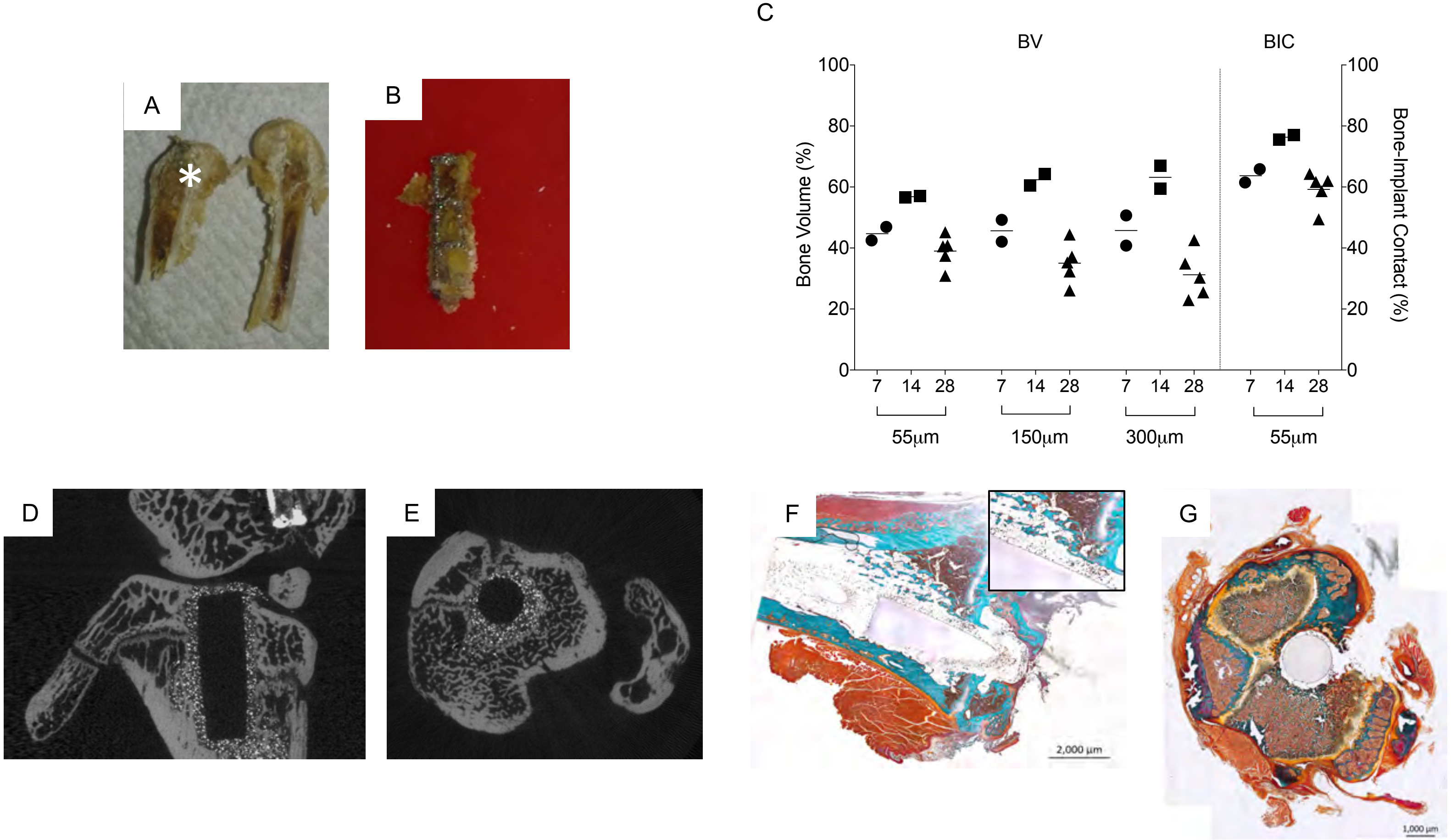

Figure S2. Representative image of A) bisected paraformaldehyde-fixed rat femur from control animal at day 14 post-surgery with titanium implant in situ (asterisk). B) Bone ingrowth was evident within the excised femoral titanium implant. C) Percentage bone volume (BV) within peri-implant region of interest set at 55mm, 150mm and 300mm distances from the implant surface and the percentage bone-implant contact (BIC) at day 7, 14 and 28 post-surgery in control animals, as determined by microCT analysis. Data show mean. Representative D) coronal and E) axial microCT scan of the cemented UHXLPE implant within the proximal tibia of a control animal at day 28 post-surgery. F) Representative Goldner trichome-stained resin-embedded F) longitudinal and G) transverse sections from an infected and control animal respectively at day 28 post-surgery showing mature bone matrix (green) and new bone formation (osteoid, red) adjacent to the bone cement mantle encasing the UHXLPE implant.

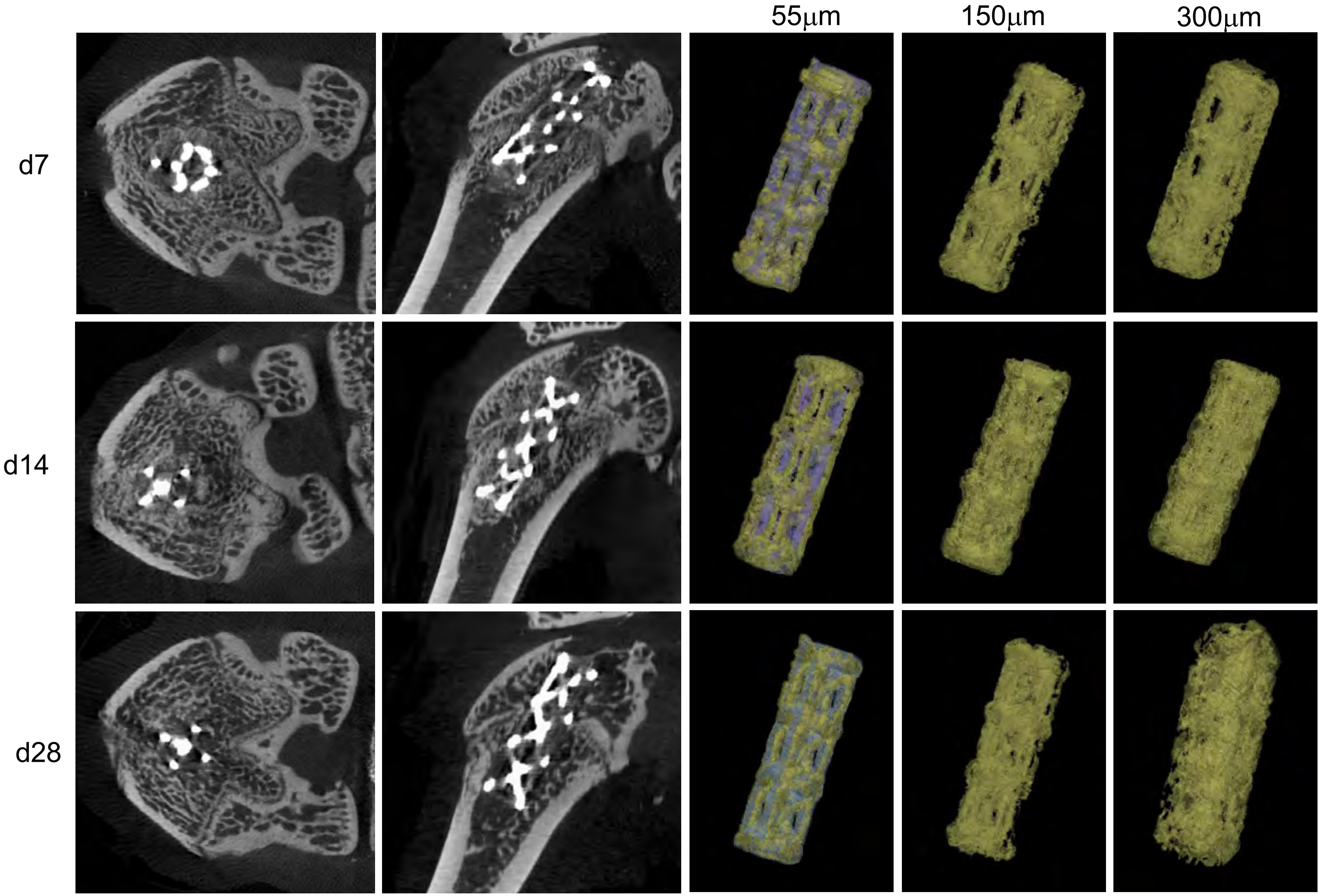

Figure S3. Representative 3D reconstructed microCT images of the distal femur of uninfected, control animals at day 7, 14 and 28 post-surgery showing bone contact and regeneration surrounding the press-fit titanium implant within cylindrical regions of interest set at 55mm, 150mm and 300mm from the implant surface (bone – yellow; implant surface – grey).

Figure S4. Representative scanning electron microscopy images of *S. aureus* ORI16_C02N biofilms grown for 24h on titanium implants under *in vitro* culture conditions, prior to (A, B; 750X and 3500X magnification, respectively) and following sonication (C, D; 750X and 2000X magnification, respectively).

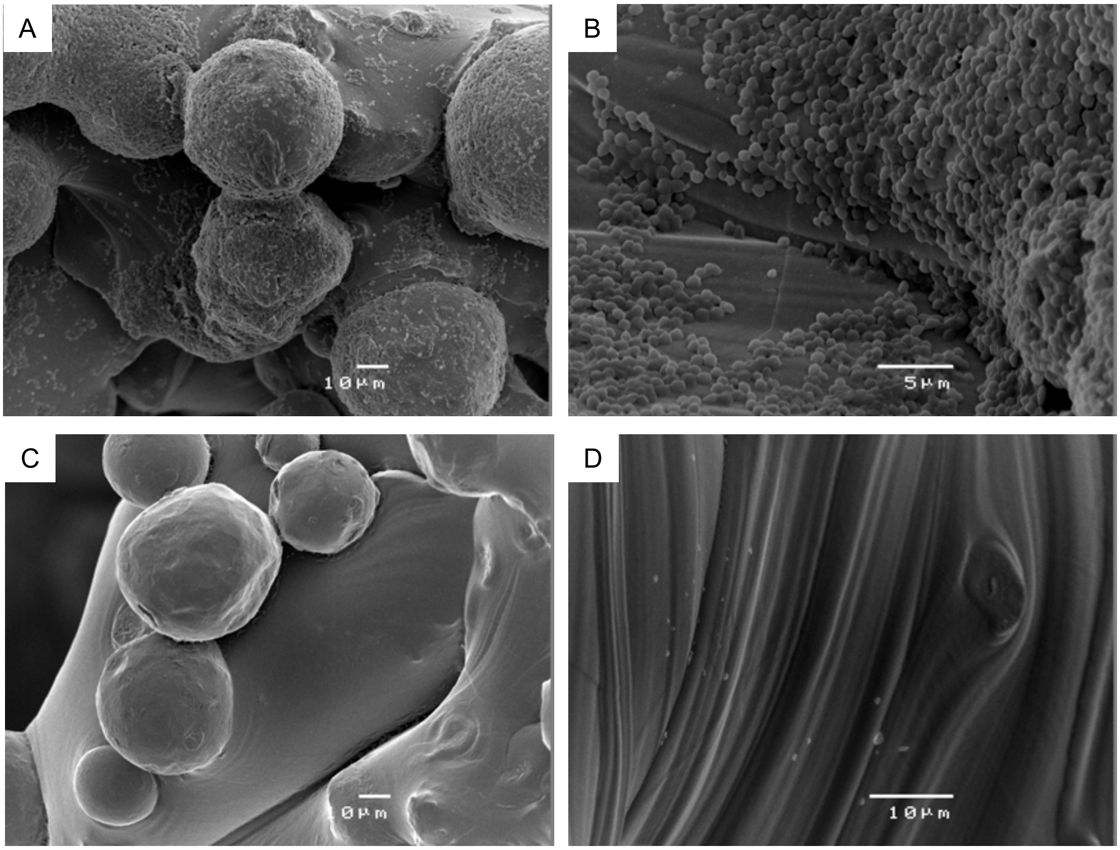

Biology Open • Supplementary information

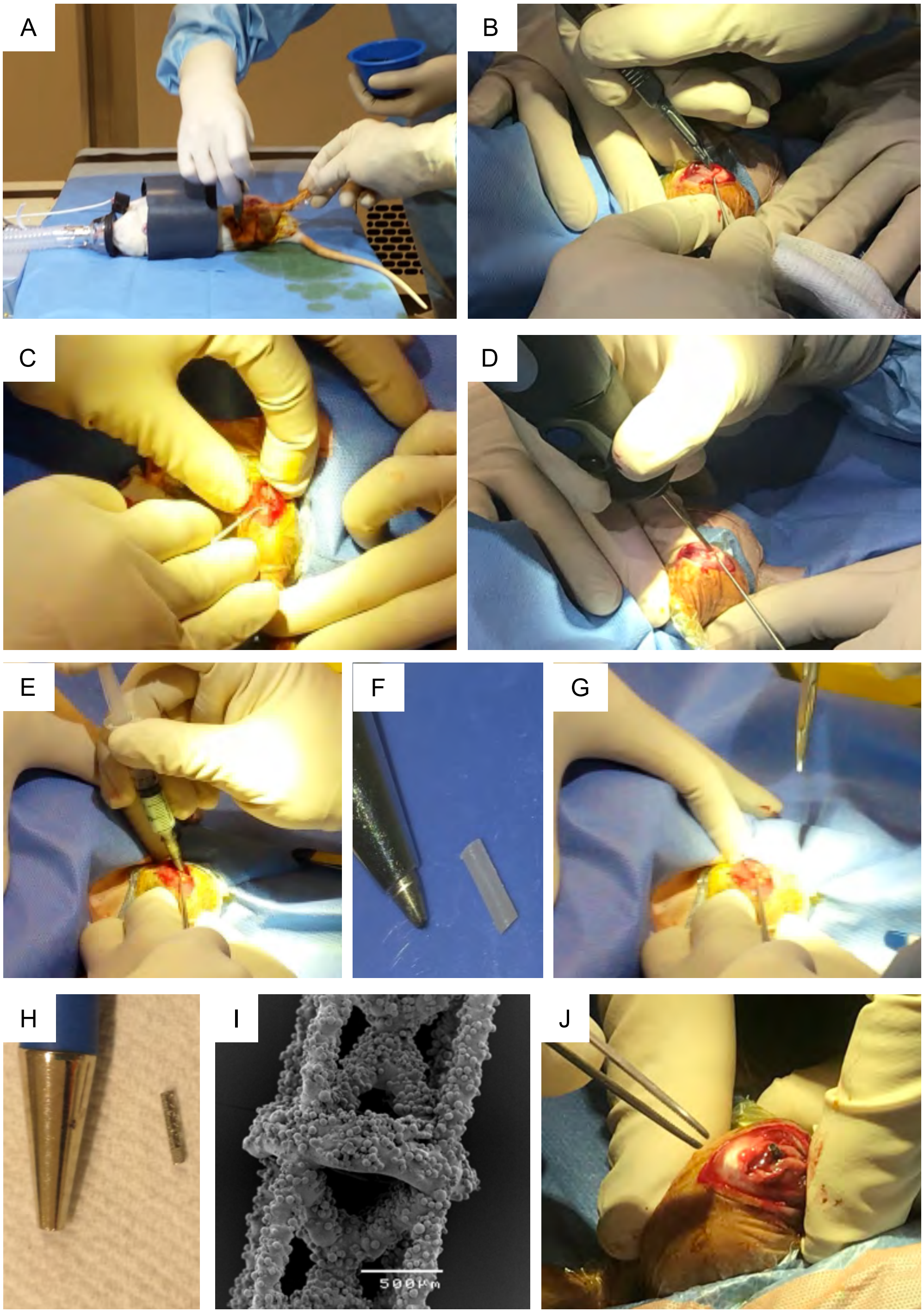

Figure S5. Surgical technique for rat model of hybrid fixation knee implant surgery. Male Sprague-Dawley rats were anaesthetised, and hair removed from the right hind leg. A) Skin was disinfected and a sterile field created. B, C) A medial parapatellar approach was used to access the articular space, with the patellar shifted laterally. D) A microdremel was used to create a defect in the proximal tibia and E) bone cement deployed using a cannula and syringe, prior to F, G) insertion of the UHXLPE implant. Following creation of an intercondylar defect in the distal femur with a microdremel, H-J) the titanium implant was press-fit into the bone using sterile forceps.

Biology Open • Supplementary information
